# Investigations on the Mechanical Properties of Conducting Polymer Coating-Substrate Structures and Their Influencing Factors

**DOI:** 10.3390/ijms10125257

**Published:** 2009-12-08

**Authors:** Xi-Shu Wang, Hua-Ping Tang, Xu-Dong Li, Xin Hua

**Affiliations:** 1 Department of Engineering Mechanics, School of Aerospace, AML, Tsinghua University, Beijing, 100084, China; E-Mails: xd-li09@mails.tsinghua.edu.cn (X.D.L.); huaxin@tsinghua.edu.cn (X.H.); 2 School of Mechanical Engineering, Nantong University, Jiangsu, 226019, China; E-Mail: tang.hp@ntu.edu.cn (H.P.T.)

**Keywords:** conducting polymer coating, microstructure feature, mechanical property, agglomerated macromolecular, cracking behavior

## Abstract

This review covers recent advances and work on the microstructure features, mechanical properties and cracking processes of conducting polymer film/coating- substrate structures under different testing conditions. An attempt is made to characterize and quantify the relationships between mechanical properties and microstructure features. In addition, the film cracking mechanism on the micro scale and some influencing factors that play a significant role in the service of the film-substrate structure are presented. These investigations cover the conducting polymer film/coating nucleation process, microstructure-fracture characterization, translation of brittle-ductile fractures, and cracking processes near the largest inherent macromolecule defects under thermal-mechanical loadings, and were carried out using *in situ* scanning electron microscopy (SEM) observations, as a novel method for evaluation of interface strength and critical failure stress.

## 1. Introduction

Conducting polymer films or coatings, such as the polypyrrole (PPy) and polythiophene (Pth), are widely used as functional, protective and decorative materials in diverse engineering applications, chemical sensors or biosensors [1–5], battery electrodes and as protection against oxidation or corrosion [6–9]. In addition, conducting polymer films or coatings are also widely exploited due to their special features such as low densities, high strength, ease of fabrication, design flexibility, stability and low cost. The promise of combining these desirable physical properties with electrical properties has prompted great interest in conducting polymers over the past few decades, and it is clear now that polymers and electrical conductivity are no longer mutually exclusive.

The search for conducting polymers began in earnest at the end of the 1970s, when an increase in conductivity by 12 orders of magnitude was observed in polyacetylene upon charge transfer doping [10]. This was closely followed by the fabrication of free standing polypyrrole films or coatings by the oxidative electropolymerization of pyrrole [11].

However, for some time, the practical applications of conducting polymers were limited by their intractability and insolubility, especially in the doped state. These problems were overcome by using 3-substituted thiophenes [12,13] and aniline derivatives [14], which not only produced processable conducting polymers, but also allowed the polymers to attain the full range of physical and mechanical properties.

Polythiophenes have been usually featured in more general reviews on conducting polymers [15–17] or on more specific topics, such as electropolymerization [18–20], electrochemistry [21] environmental stability [22], optical properties [23] and microstructure-thickness-mechanical properties [24,25]. However, so far there have been at least three reviews which have focused entirely on the synthesis, functionalization and applications of polythiophenes [26–28]. The first one was published in 1986 [26], the second—an excellent work by Roncali [27]—was published in 1992, the the third—on the synthesis, characterization and applications of thiophene-based functional polymers was published in 1998 [28]. We feel that it is timely to give an up-to-date account of functional polymers based on the thiophene family, as much progresses have been made since 1992. At the same time, these reviews have reported on the synthetic trends for the preparation of low bandgap and regioregular materials that occupy a central place in recent advances in polythiophenes, since so many of their properties are profoundly dependent on the regioregularity of the polymer, as are other special properties like chromatism and luminescence. The effect of fullerenes on the mechanical or conductive properties of polythiophene Langmuir-Blodgett films has received much attention over the past two decades, accounting for a great deal of the activity in the material science areas. Conducting polymer films or coatings however face the challenge of how to conform the internal stresses, failure models and failure behavior/mechanisms in order to meet current design requirements for practical applications, especially when the thickness of film or coating is relatively thin and relatively wide. For example, Francis *et al*. [29] studied the origins of internal stresses in polymer coatings and the available methods to measure them by using the determination of curvature or deflection of a coated elastic substrate (e.g., wafer or disk, strip, cantilever). In fact, most polymeric films or coatings shrink during and after solidification due to chemical reactions, solvent evaporation, phase separation, or some combination thereof. The strength of coating adhesion on the substrate and internal stress in the coating layers can easily cause the shrinking of the coating, leading then to brittle cracks in the coating layers or blisters from the substrate. Edge effects and thickness nonuniformities can also cloud the interpretation of stress evolution phenomena. These issues have been visited in a large body of literature [30–33]. Wang *et al*. [24,25,34] were concerned with the effects of thickness and microstructure on the mechanical properties of conducting polythiophene films, with special emphasis on failure behavior. In addition, they also demonstrated that the failure behaviors of film/coating-substrates under bending loadings or under thermal-mechanical loadings were dependent on complex factors, such as plane stress state or strain state, thickness of the layer of film and interface adhesion strength, which were in turn dependent on the synthesis processing technology used [35–39]. Baumert *et al*. [40] showed the factors affecting the forming behavior of ultra-thin polymer coatings on ductile substrates, while an extensive literature exists for the reverse case, metal films on polymer substrates, or brittle films on deformable substrates. For these cases, advanced models exist, mostly based on the so-called shear lag approximation. The heterogenous, highly localized strain and stress distributions, typical of ductile substrates, as well as out of plane displacements at the interface were disregarded, but could not be neglected at the high strain levels of interest for elastic polymer films. The role of slip bands in cracking of ultra-thin polymer films was studied. These results indicated that if the film/coating layer is thin enough relative to the thickness of a substrate, the failure behavior or model of film/coating-substrate structure may be changed. Understanding the failure transforming mechanism is still a rather complex problem and it needs to be confirmed by more experiments, although some works have found that there is a size effect. For example, the physical undulate effect on the failure model or plane stress state must also be considered. However, the most common method to determine internal stress in coating layers is only by measuring the curvature or deflection of a coated elastic substrate. This method dates back to Stoney’s work in 1909 [41]. The basic assumptions and methodologies of curvature and deflection-based measurements of stress in Stoney’s work are outlined below, and a brief discussion of other stress measurement methods and supporting characterization is presented. Relatively complete descriptions of the mechanism involved in curvature and deflection-based techniques [42–45] and reviews of internal stress measurement methods [46,47] can be found in the general literature. The internal stress in a coating may cause a deformation and result in a curvature of the system. It was argued that the failure in film/coating layers customarily might also occur in cases when the curvature or deflection of substrate might be very relatively small relative to the micro scale. Therefore, the above mentioned estimation method is very difficult or more errors exist. However, these aspects were seldom reviewed, especially with regard to the failure behavior, failure criteria stress and failure mechanisms of conducting polymer film/coating-substrate structures. Therefore, the quantificational understanding of how these issues dominate the mechanical properties of film/coating-substrate structures in reliable designs of electronic devices is an important and pivotal problem. In this review, combining both references and our own recent work, the different estimation methods, the failure behavior in thin film/coating, the cracking process and microstructural features on the meso- or micro- scale were carried out by using the SEM *in situ* technique. The effects of microstructure and the dependence on the synthesis and doping methods of the mechanical properties of conducting polymers are also characterized and discussed.

## 2. Typical Preparation and Processing of Conducting Polypyrrole/Polythiophene Coating-Substrate Structures

A polythiophene (Pth) thin film/coating was synthesized in a one-compartment cell with the use of an EG&G model 283 potentiostat under computer control. This system, which consists of working-, counter- and reference-electrodes is called a three-electrode system [48–50]. The working-electrode acts as the substrate for the electrodeposition of the Pth (or other polymers). Since the process of deposition of the conducting polymeric film/coating is an oxidative process, it is necessary that the electrode not be able to concurrently oxidize the aromatic monomer. For this reason only inert electrodes such as stainless steel substrates were used. The working- and counter-electrodes were AISI 304 stainless steel sheets (*L* × *W* × *t:* 45 mm × 2.5 mm × 1 mm) placed about 5 mm apart. The reference electrode was Ag/AgCl. The electrolyte solution was freshly distilled boron trifluoride-diethyl etherate [BF_4_]^−^ containing 30 mM thiophene at a constant potential of 1.3 V. All residual solutions were deaerated by a dry argon stream and maintained at a light overpressure during fabrication. The total charges exchanged during the deposition process were used to control the thickness of the deposited films/coating. If one wishes to obtain a solid film, the film has to be washed with distilled ethyl ether and it should be dried under vacuum at 80 °C for about 24 hours.

Another typical electrolytic solution used for polypyrrole (PPy) consists of mixtures of isopropyl alcohol (IPA, Tianjin No.1 Chemical Regent Plant, China), boron trifluoride diethyl etherate (BFEE, Beijing Changyang Chemical Plant, China), diethylene glycol (DEG) or polyethylene glycol (PEG, mw = 1,000, Beijing Yili Fine Chemical Corporation Ltd.). All solutions were deaerated by a stream of dry nitrogen for 10 minutes before the experiment and a slight nitrogen over-pressure was maintained during the experiments. The polymerization of pyrrole was performed at the ice bath temperature of 0 °C, except when indicated otherwise. PPy film/coating was grown potentiostatically. After polymerization, the PPy film/coating surface was washed repeatedly with acetone [24].

Occasionally, to enhance the adhesion strength between the polypyrrole film/coating and a substrate, the substrate should not be easily oxidized. For strongly adherent polypyrrole coatings on substrates such as smooth iron or mild steels, a treatment by 10% aqueous nitric acid that inhibits iron dissolution without preventing the pyrrole oxidation was studied [51]. Very adherent thickness-controlled PPy films were obtained at constant-current in various aqueous media containing Na_2_SO_4_, K_2_C_2_O_4_ or KNO_3_. A maximum coulombic efficiency of 95% was obtained in the presence of KNO_3_ for a current density lower than 10m A/cm^2^, but it dropped to 70% and 50%, in the presence of Na_2_SO_4_ and K_2_C_2_O_4_, respectively, and for a current density between 2 and 4 mA/cm^2^. Among the various surface pretreatments under study, the 10% nitric acid treatment was found to be the only one able to modify the surface, leading to an important passivation of the iron electrode and allowing the formation of the thickness-controlled, strongly adherent PPy films.

## 3. 3-Dimensional Nucleation Behavior of Coatings on Substrates Based on the Electrochemical Synthesis Method

**Figure 1.**
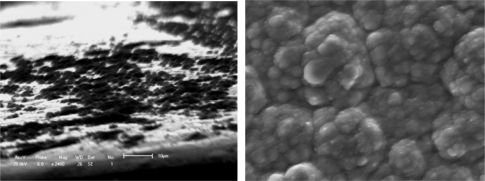
Typical deposition nucleation process of a polymer film/coating on a substrate. (a) Island microstructure of polymer [34]; (b) Surface agglomerative structure.

Figure 1 shows an evolutionary process with a macromolecular nucleation mechanism (Figure 1a) and free surface morphology (Figure 1b). This processing can be described as small island structural formation. Results are similar to most of the studies that provide evidence of nucleation and three-dimensional growth [52–54]. It is only very recently that 2-D growth has been demonstrated for electronically conducting polymers (ECP). Li and Albery [55,56] have also established the progressive nucleation and 2-D growth (2DP) for poly(thiophene-3-acetic acid). For example, Kontturi *et al*. [57] reported how the substrate material depends on the initial stages of molecular adsorption by 2-D layer by layer growth and on the high resistance of the growth film/coating. The common denominators for all these phenomena are the OH functions. C-O-C bonds are introduced in the polymer, which lead to a film with lower conductivity than normally observed for electronically conducting polymers. The low conductivity of the growing film causes the polymer to grow mainly in the direction parallel to the electrode surface (2-D growth), the potential drop between the metal electrode and the electrolyte increasing with increasing film thickness. As the amount of high resistance polymer on the electrode increases, passivation of the electrode becomes evident. The adsorption properties of the OH group are likely to contribute to the formation of an adsorbed monolayer of poly(thiophene-3-methanol) (PTM).

The layer-by-layer growth forms a compact solid structure with a certain adhesion strength, and variable conductivity of the PTM film/coating makes this polymer a possible candidate in many applications. Aside from the substrate materials, the other nucleation conditions (homogeneous nucleation or heterogeneous nucleation) can strongly influence the mechanical properties, conducting performance and microstructure feature of films/coatings. The effects on conductivity may also be significant for the protective properties against corrosion of the metallic substrate. For example, Rohwerder *et al*. recently reported a conducting polymer for corrosion protection that makes all the difference between failure and success [58]. For this purpose, Freitas *et al*. [59] and Akundy *et al*. [60,61] made great efforts to deposit or synthesize conducting polymers on different metal substrates, such as steel and aluminum. An optimization is required to balance the prevention of macroscopically extended percolation networks and ensuring enough particles in the coating to be effective. Very powerful novel corrosion protection coatings may thus be developed, based on a highly effective case sensitive inhibitor release. Therefore, the deposition process of conducting polymer on the metal substrate is similar to an island in the initial state, and it forms the thin coating layer through linking these islands reciprocally. Wang *et al*. [34] observed the certainly instantaneous morphology of deposition islands by a SEM method as shown in Figure 1.

**Figure 2.**
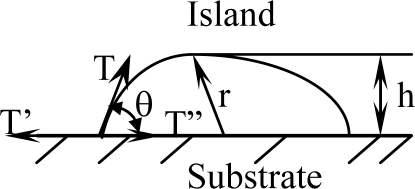
Sketch of a polymer film/coating nucleating on a substrate.

Figures 1a and 1b suggest that the deposition process of a small island morphology might be described by a nucleation model, as shown in Figure 2.

When the surface layer of island maintains a stable state, the surface tension of island is equal to the interface tension increment as follows:

(1) $$T\text{cos}\theta =T^{\prime }-T^{\prime \prime }$$

where *T* is the free surface tension needed to nucleating an island, *T*′ is a surface tension on the substrate and *T*″ is an interface tension between nucleated island and substrate. *θ* is defined as a wetting angle. The touched area between an island and substrate of initial stage can be described by Equation (2):

(2) $$A=\pi r^{2}{\text{sin}}^{2}\theta$$

and the free surface area of a formed island can be characterized by Equation (3) as follows:

(3) $$A^{\prime }=2\pi r^{2}(1-\text{cos}\theta )=2\pi rh$$

So the volume of this island formed with *A′* can be expressed by Equation (4) as follows:

(4) $$V=(4\pi r^{3}/3)[(1-\text{cos}\theta )/2]-\pi r^{2}{\text{sin}}^{2}\theta (r\text{cos}\theta /3)$$

Equation (4) can be also substituted simply by Equation (5):

(5) $$V=\pi r^{3}(2-3\text{cos}\theta +{\text{cos}}^{3}\theta )/3$$

If an island’s height is defined as *h* = *r*(1 cos*θ*) as shown in Figure 2, Equation (5) can be substituted into the relation of a wetting angle *θ* as shown in Equation (6):

(6) $$V=\pi h^{2}(r-h/3)$$

**Figure 3.**
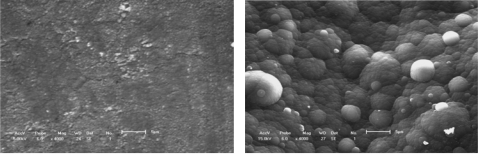
Surface structure of polymer molecular groups. (a) Conducting polythiophene coating; (b) Conducting PPy coating[24].

**Figure 4.**
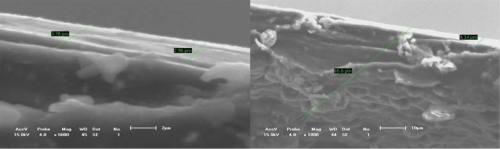
Fracture sections of conducting polythiophene film. (a) Equivalent cross-section of about 4 μm; (b) Some defects on the free surface.

The effect of all substrate materials on the nucleation behavior of conducting polymer film/coatings should be attributed to the action of the wetting angle *θ* and the interface tension magnitude during the nucleation process. However, these influencing factors depend strongly not only on the free surface quality and consistent contact between the substrate and conducting polymer solutions, but also on the electrosynthesis conditions, such as the temperature, time, electric current, *etc.* [24,57,59–61]. For instance, Kontturi *et al*. [57], Fleischmann *et al*. [62] and Harrison *et al*. [63] have described in detail nucleation models involving progressive nucleation followed by 1-D growth, instantaneous nucleation followed by 2-D growth, and 2-D growth with slow diffusion, respectively. These models have been developed for the growth of separate metal nuclei without consideration of overlap effects, and the position of electronically conducting polymers (ECP), since the deposition processes of ECPs have some differences and are usually faster than those of metals. They thought that these models of metal electrodeposition provide a good basis for analysis of the electrosynthesis of ECP, given some important clues on the nucleation and growth mechanism, and in the case of 2-D growth, on the magnitude of the spreading rate. With increasing thickness of the thin film/coating layer, polymer molecular groups are added, filling the cavities between the islands that then forming a final free surface structure of film or coating as shown in Figures 1b and 3b. Johanson *et al*. [64] gave a similar space-filling nucleation model of the planar HF/6-31G(d) optimized nitrate complex of a pyrrole pentamer dication. However, when the thickness of film/coating layer is over than 2–3 times that of *h*, the effect of substrate material on the nucleation behavior of film/coating becomes weaker and weaker indicating that there is a weak adhesive strength between the island’s interfaces. For example, the average *h* of polythiophene film/coating on stainless steel is not more than 1–2 μm, as shown in Figures 1a and 3a. Therefore, the average *h* of a PPy film/coating on stainless steel is also not more than 2–3 μm, as shown in Figure 3b. The interface or surface tension between islands may be decreased, leading easily to the formation some defects (cracks and voids) in the free surface or in the cross-section surface [25]. These defects near the free surface layer might cause the mechanical properties to gradually deteriorate, especially considering the effect of a residual stress [65] as shown in Figure 4 and the literature [24,25]. If the volume of molecular group remains a constant, with decreasing of the wetting angle *θ*, the ratio of *h*/*r* has to decrease. Then the molecular group/island on the substrate will become a more coplanar island layer. However, the work or energy of a nucleation molecular group/island can be calculated similarly to the nucleation model of metal as following as:

(7) $$\Delta \varphi =-(\pi r^{3}/3\Omega ){\left(1-\text{cos}\theta \right)}^{2}(2+\text{cos}\theta )\Delta \mu +\pi {\left(r\text{sin}\theta \right)}^{2}(T^{\prime }-T^{\prime \prime })+2\pi r^{2}(1-\text{cos}\theta )T$$

where (*πr*^3^ / 3Ω)(1 cos*θ*)^2^ (2 +cos*θ*)Δ*μ* is the free energy of nucleation molecular group/island and the minus sign means the free energy is decreasing. Ω is a molecular volume. Δ*μ* is a reduced free energy of molecule during liquid-solid transformed processing. *π* (*r* sin*θ*)^2^ (*T*′-*T* ″) is a free surface energy change value between the substrate and a touched island of molecular group. 2*πr*^2^ (1 – cos*θ*)*T* is a surface energy change value of an island. Therefore, when the critical radius of a nucleation island is a maximum value of *r_c_* = 2Ω*T*/Δ*μ*, the nucleation work of a conducting polymer can be also expressed by Equation (8) because it is similar to the deposition mechanism as follows [66]:

(8) $$\Delta \varphi_{c}=(16\pi \Omega^{2}T^{3}/3\Delta \mu^{2})f(\theta )$$

In addition, Beck *et al*. [67] have also given an estimation method for the effective thickness of polymer films on iron substrates as follows:

(9) $$h=\frac{\gamma Q_{A}m_{e}.th}{\rho }=\frac{Q_{A}(M_{m}+ym_{A})}{\rho F(2+\gamma )}$$

where *Q_A_* is the consumed electrical quantity, *γ* is the reaction coulombic yield, *M_m_*, *M* *_A_* are respectively the masses of a pyrrole repeat unit and of the doping ions, *y* is the doping yield calculated from photoelectron (XPS) spectra measurements, *ρ* is the polymer relative density at 20 °C, *F* is Faraday constant. That is, the thickness of a coating on a substrate can be calculated according to the electrochemical synthesis conditions. These studies confirmed that the effect of substrate materials on the deposition thickness of polymer cannot be ignored. The effect factor can be determined from whether inside and surface defects or the effective fracture cross-section.

## 4. Microstructure-Mechanical-Conductive Properties of Polymer Film/Coating

Based on the above mentioned deposition models of electrochemically synthesized conducting polymer film/coatings [57,62–64], the microstructure of the free surface or fracture cross-section depends strongly on the synthesis conditions. The perfect microstructure of a conducting polymer should satisfy the requirements of mechanical and physical properties such as stiffness, mass and temperature. For example, Chainet *et al*. [68] suggested that the polypyrrole morphology under different doping states should be studied *in situ* with scanning tunneling microscopy (STM). Their results indicate that the electronic conducting polymer was submitted to strongly structural variations during the doping (or dedoping) process due essentially to the inclusion of anions in its structure. The insertion of ions induces a swelling phase and therefore modifies the surface morphology. They obtained an evolution of the cross-section profile of the polymer strands as a function of the potential applied to the thin film. Surface roughness of conducting polymer coating layer was found to be rather low in comparison to other polypyrrole salts, even at thicker layers [67]. The change of morphology caused necessarily a change of the mechanical properties of the conducting polymer. This is because the morphology change may cause a structural defect or a geometrical notch. For the surface morphology and defects capacity per unit area of conducting polythiophene film, Zuo *et al*. [69] suggested that the fractal characteristic of surface morphologies and defects capacity in a unit area might be described by using fractal theory. Fractal results are indicated to be of value in the quantitative characterization of surface morphologies and defects capacity per unit area of electrochemically synthesized polythiophene films. The fractal characterizations of surface morphology and defects capacity in the unit area depended mainly on the grizzly threshold on these SEM images. The relationship between the threshold and the fractal dimensionality can be expressed as a function. The fractal dimensionality is as follows:

(10) $$D=n-\frac{A}{1+\text{exp}(\alpha +k)}$$

where *n* is a fractal ideal dimensionality (2 or 3), *A*,*α* are the fitting constants, *k* is the gray threshold value on the SEM image of free surface microstructure. The fractal dimensionality of the surface morphology and microstructure of these films might be related to the mechanical properties/behaviors or their electrochemical synthesis processing. At the same time, Cassugnol *et al*. [70] studied the microstructure-conductivity relationship in conducting polypyrrole/epoxy composites. They classified four dispersing methods, which were used to disperse increasing amount of PPy, synthesized either via dispersion (PPy_d_) or suspension polymerization, in an insulating epoxy matrix in order to ensure that it has a good electrical conductivity. These results indicated that while the classical percolation theory predicted in the case of 3-D system a percolation threshold of 16 vol% of filler concentration, none of the blends elaborated met this theoretical value. In fact, their predication was based on the assumption of the presence of spherical, isotropic, monodispersed and randomly dispersed filler particles in the matrix. The influence of filler morphology on the percolation threshold was studied. Although Lux [71] also reported that small particles lead to a lower percolation threshold, this was not confirmed by the present studies, and conductive PPy exhibiting larger particle sizes leads to blends which show the lowest percolation threshold. The smaller particle size easily increases the changed tendency to agglomeration. Therefore, in the blends prepared with the PPy exhibiting the smallest particles size, *i.e.*, PPy synthesized by dispersion polymerization, a great fraction of the small PPy_d_ particles form agglomerates. These large agglomerates become the conductive entities and consequently, a high PPy concentration is necessary to form an infinite cluster which ensures the electrical conduction throughout the blend. According to the analysis in the literature [70], the PPy prepared via suspension polymerization is the most convenient filler to make the epoxy matrix electrically conductive. The larger agglomerates present in the PPy_d_/epoxy matrix can act as the initiating flaws, and may give rise to poor mechanical properties. In addition, Mano *et al*. [72] and Xu *et al*. [73] studied the thermal, mechanical and electrochemical behavior properties of poly(vinyl chloride)/polypyrrole (PVC/PPy) blends. High-strength and highly-conductive polymer films could be obtained based on electrochemical methods. They characterized the products by attenuated total reflectance FTIR spectroscopy, differential scanning calorimetry and dynamic mechanical analysis. Infrared reflectance spectra suggested that the polymerization occurred preferentially on the matrix surface producing sandwich-type structures. The mechanical, thermal and conducting behavior showed a dependence on the initial concentration of FeCl_3_ in the matrix and time of exposure to pyrrole vapour. In addition, the stress-strain behavior of PVC/PPy blends were obtained from PVC/FeCl_3_ matrices and exposed to pyrrole for 2 h and 6 h indicated the important changing trend that the yield stress and Young’s modulus of film/coating decreased as the FeCl_3_ increased from 1% to 35%. The elongation at break of the film/coating increased slightly as FeCl_3_ was increased from 1% to 35%. Wang *et al*. [24,34,74] discussed the relation of microstructure-mechanical properties in conducting polypyrrole films. Their results validated the relationship between the conductivity/mechanical properties of PPy films and synthesis conditions, as shown in Table 1. Therefore, the influence of synthesis temperature on the mechanical properties of a conducting polymer is obvious, but that on the conductivity of a conducting polymer is not obvious. In addition, the influence of the added ions on the conductivity of conducting polymer is also very obvious but that on the mechanical properties is not obvious. However, the effect of thickness on the strength of the film is very obvious because the strength (and Young’s modulus) of the film changes with increasing of the film thickness from 4 micrometers to 10 micrometers [25]. In order to describe the effect of microstructures on the tensile strength and brittle-tough properties of the PPy films/coating, a typical example was used as one case of BFEE concentration (5%, by value) to analyze the relation and the effect of the temperatures and PEG concentrations on the mechanical properties were discussed (the conduction polymer is covered in the next section in detail).

**Figure 5.**
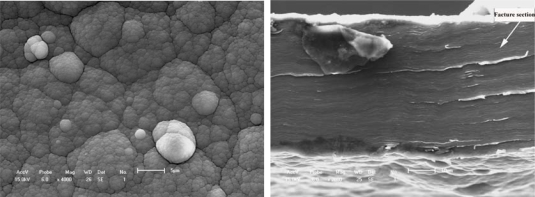
Free surface morphology and cross-section fracture of PPy film synthesized in the electrolyte of IPA + 5% BFEE + 5% PEG1000 at 15 °C. (a) Surface morphology (b) Cross-section fracture

**Figure 6.**
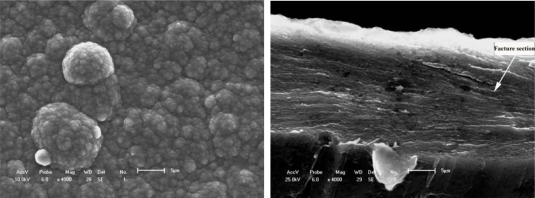
Free surface morphology and cross-section fracture of PPy film synthesized in the electrolyte of IPA + 5%BFEE + 5%DEG in an ice bath at 0 °C. (a) Surface morphology [24] (b) Cross-section fracture [24]

Figures 5 and 6 show the typical free surface morphology and cross-section fracture of conducting polymer films which were prepared in mixed electrolytes of IPA + 5% BFEE in an ice bath at 15 °C and 0 °C respectively. Figures 5a and 5b show the free surface and cross-section fracture of PPy film synthesized in an electrolyte of IPA + 5% BFEE + 5% PEG1000 at 15 °C, respectively. The results indicate that there are rather larger and flatter agglomerated groups and rather more grooves on the free surface, which forms a smooth cross-section surface of fracture as a result. The characterizations confirm the film has a poor toughness, whose elongation at break is about 4.47% and its tensile strength is about 36 MPa, as shown in Table 1. This is because the adhesion strength among the agglomerated groups is rather low. The surface cohesive force of this microstructure may be weaker than that of cap shape aggregation structure formed by capillarity or island microstructure formed by 3-D nucleation.

Figures 6a and 6b show the typical free surface and cross-section fracture of PPy film synthesized in an electrolyte of IPA + 5% BFEE + 5% DEG in an ice bath at 0 °C. Tensile strength and toughness were improved in an ice bath at 0 °C, especially the tensile strength of the conducting polymer. In view of surface morphology either free surfaces or fracture cross-sections as shown in Figures 5 and 6, there are some differences. For example, there are less grooves in Figure 5a than in Figure 6a. This means that there is a rather stronger aggregation structure in Figure 6a than in Figure 5a, and that there is more roughness in Figure 6b than in Figure 5b. Therefore, the mechanical properties and microstructure as shown in Figure 6 are better than those in Figure 5, where the tensile strength is about 60.30 MPa and the elongation about 6.38% as shown in Figure 6, and the tensile strength is about 30.10 MPa and the elongation is about 4.47% as shown in Figure 5. The relative increments of tensile strength and elongation under different synthesis conditions reach 67.04% and 42.73%, respectively.

In order to enhance the strength of film, the focus of most studies is the application of methods of doping ions, fibers and particles into the matrix. These studies have been greatly promoted in the recent several decades. For example, Tjong [75] investigated how to reinforce the mechanical properties of polymers with lower volume fractions of nanocomposites, carbon nanotubes. Tjong described that the mechanical properties with nanocomposites depended greatly on the chemistry of the polymer matrices, nature of nanofillers, and the way in which they are prepared. The uniform dispersion of nanofillers in the polymer matrices is a general prerequisite for achieving the desired mechanical and physical characteristics. He also described in detail current developments on the processing, structure, and mechanical properties of polymer nanocomposites reinforced with layered silicates, ceramic nanoparticles and carbon nanotubes, respectively. In addition, Somanathan *et al*. [76] and Andreatta *et al*. [77] suggested that both the conductivity and tensile strength of a polymer were strongly affected by both inter- and intra-chain interactions. However, after comparison of the conductivity results of P3c6T with P36T, Moulton [78] expressed that the effect produced by drawing is very pronounced in the case of P3c6T. The mechanical and morphological aspects of P3c6T at different temperatures clearly showed the brittle-ductile transition that taken place in the material and the film formed into a compact structure. In addition, Wang *et al*. [79] analyzed the effects of the addition of amounts of montmorillonte with different doping nanocomposite particles on the mechanical properties of polyimide nanocomposite films based on the cross-section fracture analysis of the film. The incorporation of montmorillonite nanolayers into the polymer matrix can results in an evident enhancement in the Young’s modulus, but the elongation decreases. The Young’s modulus increase is almost solely attributed to the reinforcing effect of the dispersed montmorillonite nanolayers. The analysis of fracture morphology also showed also that the incorporation of 1 wt% montmorillonite content produced the best tensile behavior. Therefore, the improvement of mechanical properties could be ascribed to the uniform exfoliation and dispersion of the nanolayers and enhancement of interfacial strength which depended on the size of the composite sheet between the montmorillonite content and matrix phases. At the same time, Pettarin *et al*. [80] reported the relation of morphology and mechanical properties of polyethlene/(organo-montmorilloite) composites modified with ethylene/methacrylic acid copolymer. All polyethlene/(organo-montmorilloite) composite morphologies were examined using X-ray diffraction (XRD), scanning electron microscopy (SEM) and transmission electron microscopy (TEM) technologies. The mechanical properties were evaluated under static conditions. A slight reinforcement was achieved only when the organoclay montmorillonite (OMMT) was added to polyethylene (PE). The addition of a small amount of ethylene methacrylic acid copolymer (EMAA) to the composite films could result in improvements of the level of exfoliation [81,82], but there is no positive effect on exfoliation when EMAA was added to the composite morphology. XRD patterns of PE/OMMT/EMAA exhibited a peak with lower *d*-spacing than OMMT. Uniaxial tensile tests and heat distortion temperature (HDT) followed the same tendency: as EMMA was added to the system, the properties of composites deteriorated approaching the level of the pristine matrix. Also, SEM micrographs provided some evidence of OMMT encapsulation by EMAA. Jordan *et al*. [83] have reviewed the recent work polymer matrix nanocomposites. In their review, they provided an overview of the processing technology and trends in the mechanical behavior and morphology of nanocomposites. In general, the mechanical properties of polymer nanocomposites are superior to those of the pure polymer matrix or composites with larger sized inclusions or other defects such as voids. The effects of the nanoparticles are dependent on many variables parameters, especially upon the relative crystalline or amorphous nature of the polymer matrix, and the interaction between the filler and matrix. As for the effects of micro-inclusions or micro-voids on the mechanical properties of materials, Wang *et al*. [84–90] have provided a significant amount of SEM *in situ* observation results. Although the results referred to metals with micro-inclusions or fatigue problems, the investigation method and effects of the mechanism of inclusion on the mechanical properties of materials are still applicable to the characterization of polymers filled with other materials. These results indicated mainly that the mechanical properties, including to the fatigue properties, are not only dependent on the added amount of particles, critical size, shape, types of particle and the ratio of hardness level between particle and matrix, but also on the obliquity between the longest axis of particle and applied loading direction, especially for a rectangular hard inclusion or doping of hard particles. This effect is most dangerous when the angle between the longest axis of the particles and applied loading direction is about 90°. This is because micro-cracks then occur easily at the interface between particle and matrix, and the crack easily propagates along from the interface to the matrix, even if the adherence strength between the particle and matrix is great enough.

Kaynak *et al*. [91] demonstrated the changes to the mechanical and electrical properties of polypyrrole films with concentrated dopants and oxidative aging. They described the process of mechanical and electrical properties loss for up to 12 months of aging in air at room temperature. The results indicated that the tensile strength of films highly doped with *p*-toluenesulphonate was lower than that of lightly doped ones. The difference was attributed to the presence of stress concentrations on the nodular surfaces of the films with high concentrated dopant. Highly doped films were electrically more stable, with a lower rate of loss of conductivity with time, compared to lightly doped ones. The conductivity of the latter initially decreased at a different rate from that observed after a prolonged time, exhibiting two distinct aging behavior regions. In contrast, highly doped PPy films followed first-order kinetics with a very small loss of conductivity over the aging period. The conductivity decline rate was dependent on aging time, dopant type, and concentration, resulting in a complex conductivity decay mechanism.

**Figure 7.**
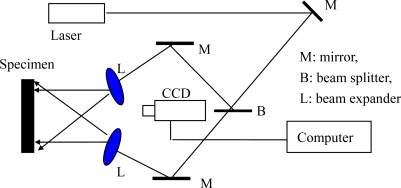
Schematic figure of the deformation measuring system [92].

**Figure 8.**
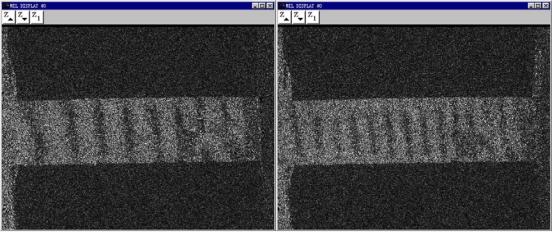
Electronic speckle pattern interferometry images under different loadings [92]. (a) F=2.63 N (b) F=3.53 N

As another mechanical property parameter of conducting polymer film/coatings, the elastic modulus of the conduction polymer is very important in its applications, such as microelectronics, energy storage, electrocatalytic systems, photovoltaic devices, electrochromic devices, *etc.* In the case of conductive polymers potentially useful in electrical applications, the elastic modulus is a key mechanical parameter in the above mentioned applications. However the process of accurately measuring the mechanical parameters of a polymer is rather difficult. This is because how its constitutive stress-strain equation is obtained depends directly on its reliability, but the deformation of high flexibility films cannot be measured by the general methods. Wang *et al*. [25,35,39,78,92,93] used an electronic speckle pattern interferometry (ESPI) technology [94,95] and the digital image correlation (DIC) method [96,97], respectively, for measuring the deformation of highly flexible specimens of conducting polymer. Figure 7 shows a typical electronic speckle pattern interferometry system for measuring the in-plane displacement of a free surface by electronic speckle pattern interferometry (ESPI) technology. The laser beam is reflected from a mirror, and then split into two beams with a splitter. One is expanded by a spatial filtering setup and is used as the object beam illuminating a diffuse object; and the other is reflected from a mirror and is expanded by another spatial filtering setup, forming the reference beam. A zoom system is used to focus the object onto the sensor of a CCD camera. Thus, each sensor receives the coherent superposition of the object beam and the reference beam. The interference intensity distribution of a subtractive speckle correlation fringe pattern can be expressed by Equation (11):

(11) $$|I^{\prime }(r)-I(r)|=\left|4u_{0}u_{R}\text{sin}[(\varphi_{0}-\varphi_{R})+\frac{\Delta \varphi (r)}{2}]\text{sin}(\frac{\Delta \varphi (r)}{2})\right|$$

where *I*′(*r*) and *I*(*r*) are the speckle intensity distributions before and after object deformation, respectively, *u*_0_ and *u_R_* are the intensity distributions of the object and reference beams, *ϕ* and Δ*ϕ* are the random phase of the speckle distribution and the phase variation due to the object deformation. The relationship between Δ*ϕ* and displacement is expressed by Equation (12):

(12) $$\Delta \varphi =\frac{2\pi }{\lambda }[W(\text{cos}\theta -\text{cos}\theta )+U(\text{sin}\theta +\text{sin}\theta )]=\frac{4\pi }{\lambda }U\text{sin}\theta$$

where *λ* = 694.3 nm is the wavelength of laser, *W* is the out-plane displacement, *U* is in-plane displacement component normal to the surface of polymer film, and *θ* is the angle between the illuminating and the viewing directions. By using the two beams measuring system as shown in Figure 7, the out-plane displacement is eliminated and Δ*ϕ* is sensitive to the in-plane displacement *U.* When Δ*ϕ* = 0 or *nπ,* the displacement *U* in Equation (12) may be replaced by the displacement increment Δ*U* along the *y* -axis direction (the longitudinal direction of the sample as shown in the typical images of Figure 8). Then, the displacement of the *y*-axis direction can be expressed by Equation (13):

(13) $$\Delta U=\frac{n\lambda }{2\pi \text{sin}\theta }$$

where *n* is the number of fringe patterns in the direction of *y*. Therefore, once the number of fringe patterns in the *y* direction is known, the deformation in this direction can be determined from the the Equations (11–13) above.

**Figure 9.**
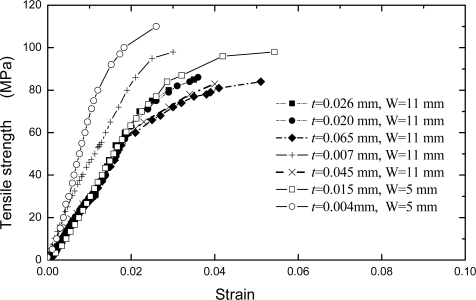
Stress and strain curves of Pth film with the different thicknesses [25].

A rectanglular sample of Pth film usually undergoes considerable shape changes as it is subjected to *L* even a small external force. The change takes place mainly lengthwise because of the ratio $\frac{L}{W}>10$, where *L* is the length of the sample, and *W* is the width. The micro-change in the *y* direction, which is subjected by the applied loading direction, can be detected by the ESPI system. About 12 fringe patterns of the Pth beam under different the applied loading levels were recorded. Two typical fringe patterns are illustrated in Figure 8. It is clear that the significant fringe patterns are displayed. According to the number of the fringe patterns, the amount of deformations of Pth film in the applied loading direction can be calculated easily by the above equations. The engineering stress-strain curves of Pth films of different thicknesses are shown in Figure 9.

Following the linear elastic deformation, plastic flow commences at a stress approximately equal to the yield stress, *σ* _0.2_, defined as the stress corresponding to a strain of 0.002. During plastic deformation, an evident strain-hardening is observed, that is, the stress required increases with the development of plastic strain. The maximum strength of a conducting polymer film is approximate to that of aluminum thin film when the thickness is smaller than about 10 μm. It can be seen from Figure 9 that the strengths of Pth films varied with the thickness. That is, the tensile strength increases as the Pth film thickness decreases. The strengths of films are especially sensitive to the film thickness when the latter is less than about 10 μm. However, the strengths of films approach a constant value when the film thickness is more than about 20 μm. Especially, an evident change has been observed in *σ*_0.2_ when the thickness is more than about 20 μm. A film with the thickness of 4 μm possesses the highest strength. This is because the thin film/coating early stage of deposited nucleation process is strongly influenced by substrate so that the thickness forms a correspondingly compact structure, but with the increasing of thickness, this influence becomes smaller and smaller. Therefore, the defects and cracks easily occur at the relative thicker film/coating layer. This insufficiency of defects and cracks in film/coating layer can be improved by the appropriate synthesis methods [18,24,45,51,67,73–82].

**Figure 10.**
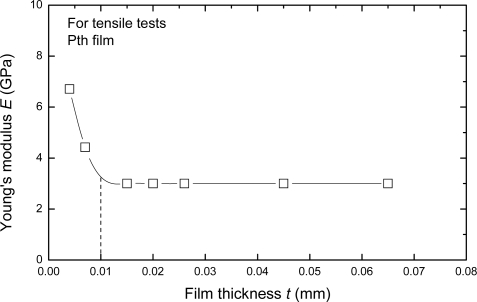
The relationship between Young’s modulus and film thickness [25].

The elastic (Young’s) modulus also varies pronouncedly with the thickness of film which is less than about 10 μm, but it approaches a constant when the thickness is more than about 10 μm. The relationship between elastic modulus and the thickness is shown in Figure 10. The main reason of this phenomenon seems to associate with the growth mechanism and its influence factors of the Pth films. When the thickness is less than 10 μm, the Pth mainly grows up in a stable manner on the base layer. But with increasing thickness, more and more agglomerated molecule groups are formed away from the base layer. Therefore, many micro-defects such as micro-cracks and micro-voids easily exist in the adjacent surface layer. At the same time, the binding energy of these agglomerated molecule groups becomes smaller and smaller because of the electrochemical influence or the influence of the metal substrate. Therefore, the microstructures of Pth films will change as the thickness increases. A thin film with the thickness of about 4 μm generally has a perfect microstructure with few defects.

In most applications, the film/coating-substrate structure appears in the components and devices. The estimation of the mechanical properties of the film/coating-substrate system has been investigated over the last several decades [41,98–102]. These mechanical properties of the film/coating-substrate structure refer to the many influencing factors such as internal stress, adherence force and the elastic properties of the thin film. Using either the static bending [41,98,100] or the vibrating reed technique [99,101] considering the integrated structure, the Young’s modulus *E_f_* of film-substrate structure can be estimated by the vibrating method:

(14) $$E_{f}=\frac{E_{s}}{3}(\frac{\rho_{f}}{\rho_{s}}+\frac{2d_{s}}{d_{f}}\frac{\omega -\omega_{0}}{\omega_{0}})\frac{1-\nu_{f}^{2}}{1-\nu_{s}\nu_{f}}$$

where ω,ω_0_ are the resonant frequencies of the coated and uncoated samples, respectively, *ρ* is the density, *d* is the thickness and *ν* is the number of lateral contraction, where subscripts *s*, *f* are substrate and film/coating, respectively. Since the result in the Equation (14) is very sensitive to the change of frequency, a small effect on the frequency must be taken into account as well. Therefore, the estimation value of *E_f_* in Equation (14) is more suit for thin layer on the metal substrate. And the estimation value of *E_f_* based on the static bending method [41] is more suit for the thick layer on any substrate. In addition, there is rather greater viscous–elastic ratio in the polymer film/coating layer and metal substrate. It is difficult to obtain the resonant frequencies in the Equation (14). Therefore, the estimation method of Young’s modulus and strength of film/coating-substrate structure are generally using the mechanical method. The intrinsic or applied stress in thin film/coating layer cause curvature (expressed by the radius *R*) of the sample which can be calculated for thin film/coating-substrate from Stoney’s formula [41]:

(15) $$\sigma_{f}=\frac{d_{s}^{2}E_{s}}{6Rd_{f}(1-\nu_{s})}$$

Though the determined curvature (or bending) method can estimate the stress in film/coating layers, the internal stress experimentally measured by the curvature of a sample has even more errors. Stoney’s linear formula is most commonly used, but, it may be inadequate owing to nonlinear effects that should not be ignored. For example, taking into account the case of a rectangular plate, the role of the different parameters controlling the geometrical nonlinear effects are examined, and then the one-to-one mapping that exists between the degree of nonlinearity and the parameter *b*^2^ / *d* _s_ *R* (*b*,*d_s_*, *R* are the sample’s width, thickness and bending curvature, respectively) can be established [100]. In estimations of the internal stress of film/coating through the curvature method, important nonlinear effects may affect the validity of Stoney’s formulae. Townsend *et al*. [103] and Fawcett *et al*. [104] modified Stoney’s formula and measured the Young’s modulus of thin films on thick substrates by a novel method. Stoney’s formula accepts that curvature may cause some errors, especially when the substrate is difficult to inflect. Therefore, this effect of flexural and torsional modes must be corrected by stress measurements for accurate determination of the thin film/coating substrate structure [99]. Besides, with narrow cantilevers the frequency is very sensitive to curvature [102]. The ratio of frequencies of the torsional and flexural modes mainly depends on the curvature. Thermal expansion and residual stress are two important mechanical parameters of conducting polymer coating-substrate structure or of microelectronics and microelectromechanical systems (MEMS). There are several problems in that the mismatch of thermal expansion between the thin film/coating and the substrate may lead to residual stresses in thin film or coating layer reported in literature [105]. The residual stress will cause damage or deformation mismatch in thin film/coating- substrate structures [65]. Fang *et al*. [106] found that the coefficient of thermal expansion (CTE) of thin films varied with the changing of the thickness on the micro scale. These results indicated that thickness of thin film/coating influenced not only the strength and Young’s modulus but also the CTE of thin film/coating layer. However, the thickness of thin film or coating layer is too thin, causing difficulties in determining these mechanical parameters by a general method. The novel methods for measuring mechanical properties are necessary, such as the bilayer microcantilever technique, the scratch and SEM *in situ* observation methods, *etc.*

## 5. Micro Cracking Behavior of Macromolecular Coating-Substrate Structure under Thermal-Mechanical Loadings

To estimate the thermal contribution of the film/coating-substrate structure in the equivalent stress formula, it should know an effective behavior of the different thermal expansion coefficient on the crack initiation or mechanical properties worsen. The cracking tests under thermal-mechanical loading were carried out from 30 °C to 100 °C. The applied thermal loading ratio is a 2.67 °C/min then holding 1 hour in the vacuum chamber of SEM using a specially designed servo-hydraulic testing system. The temperature control is by a thermoelectric couple and it has a precision of about ±1 °C. Given the fixed tension stress is 73 MPa for AISI 304 stainless steel substrate during the applied thermal loading, the relationship between the displacement increment Δℓ of free surface film/coating layer, temperature and linear thermal expansion coefficient can be defined as follows when the interface strain mismatch between the film/coating and substrate can be ignored:

(16) $$\alpha =\frac{\Delta \ell }{\ell \Delta T}(/{}^{\circ}\text{C})$$

where ℓ is the total deformation length (20 mm) of sample. Δ*T* is a temperature increment of sample. Therefore, the thermal expansion coefficient of the coating-substrate structure can be determined. For the average thermal expansion coefficient of coating-substrate structure is about 15 × 10^−6^ /°C less than that (18 × 10^−6^/°C) of substrate of AISI 304 stainless steel, the thermal stresses of the coating layer and that of the substrate structure can be simply given as following in, respectively:

(17) $$\sigma_{th-f}=E_{f}\alpha_{f}\Delta T\sigma_{th-s}=E_{s}\alpha_{s}\Delta T$$

**Figure 11.**
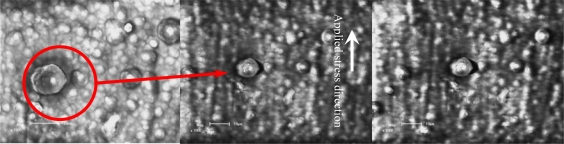
Cracking mechanism of conducting polythiophene under thermal-mechanical loadings based on SEM *in situ* observation (scale bar 10 μm). (a) Heating up from 30–100 °C; (b) Holding 15 min at 100 °C; (c) Holding 50 min at 100 °C.

Figure 11 shows the crack initiation development of a film/coating-substrate structure based on SEM *in situ* observation under different temperatures and holding times. To observe the crack initiation or propagation behavior, the special zone on free surface of polythiophene film/coating was chosen as a fixed observation zone, as shown in Figure 11a. Comparing the images in Figures 11a and 11b, it was seen that the effect of thermal-mechanical loading on the crack initiation near the interface of a molecular grain on the free film/coating surface is very obvious. It can be seen that the crack open displacement (COD) of 2.80 μm exists near the interface of the agglomerative molecular during the applied and held temperatures. At this moment, the estimated thermal stress reaches about 199.50 MPa, based on Equation (17). It can be deduced that this stress value is big enough to laniate the film compared with the failure strength as shown in Figure 9. Therefore, the estimated value by the Equation (17) is not reliable. However, if we assume there is a consistent deformation of both the film and substrate, it is possible to calculate the stress in the film layer via the elastic strain and Young’s modulus of substrate. The elastic strain is about 0.13% when the strength of the substrate is about 199.50 MPa. If the strain of the free surface of the film/coating layer is also about 0.13%, the interior stress in the film/coating layer at the strain of 0.13% is approximately equal to about 6–10 MPa, according to the stress-strain curves relation as shown in Figure 9. Therefore, the difference of stress values based on the estimation methods refers to rather complicated scientific issues, which should be studied further. However, the interface COD can reflect the fact that the maximum displacement trend is about 90° tilted to the applied mechanical loading direction, and another is that the crack occurs first at the maximum agglomerative molecular gain (its diameter is about 13.00 μm) in the view-scale as shown in Figure 11b. Figure 11c shows the crack propagation behavior, which is along a direction tilted about 90° with respect to the applied mechanical loading direction and the interface crack does not occur in another direction (the parallel applied mechanical loading direction) even if the thermal stress can be defined as 2-D or 3-D stress states. With an increased holding time at 100 °C, the COD increases quickly from 2.80 μm to 3.12 μm. It should noted that the size and shape (about 13.00 μm) of this molecular gain is almost unchanged compared with Figures 11a and 11c. The driving force for an interface crack at various depths *a* / *d* *_f_* (*a* is the crack depth, *d* *_f_* is the diameter of this grain) has been described by Hutchinson and Suo [107]. These results indicated that the dimensionless stress intensity factor, $K/\sigma \sqrt{d_{f}}$ where *K* is a stress intensity factor), depends only on the relative crack depth, *a* / *d* *_f_*, and Dundurs’ parameters [108], *α* and *β* as follows:

(18) $$\begin{array}{l} \alpha =\frac{\mu_{1}(\kappa_{2}+1)-\mu_{2}(\kappa_{1}+1)}{\mu_{1}(\kappa_{2}+1)+\mu_{2}(\kappa_{1}+1)}\\ \beta =\frac{\mu_{1}(\kappa_{2}-1)-\mu_{2}(\kappa_{1}-1)}{\mu_{1}(\kappa_{2}+1)+\mu_{2}(\kappa_{1}+1)} \end{array}$$

where *κ_i_* =(3-*ν_i_*) /(1+*ν_i_*), *κ_i_* =3–4*ν_i_* are constants in the plane stress and plane strain, respectively. μ*_i_*, *ν_i_* are the shear modulus and Poisson’s ratio of film/coating and substrate materials, respectively. For a small ratio of *a* / *d* *_f_*, regardless of the elastic mismatch, the stress intensity factor merges to that of an edge crack in a semi-infinite space, *i.e.*, $K\to 1.1215\sigma \sqrt{\pi a}\;\text{as}\;a/d_{f}\to 0$. Asymptotic behavior for another limiting case, *a* / *d* *_f_* →1, can be obtained by invoking the Zak-Willians singularity. That is, the stress singularity for a crack is perpendicular to, and at the tip or, the interface. Instead of the the square root singularity, the stresses near crack tip can be described by *σ_ij_* ∼ *K̃r*^−1^ *f_ij_* (*θ*), where (*r*, *θ*) is the polar coordinate centered at the crack tip, and *f_ij_* are dimensionless angular distributions. The scaling factor *K̃* plays a part analogous to the regular stress intensity factor, but has the different dimensions. Therefore, the phenomenon of microcrack propagation showed that the failure of film/coating-substrate structures depends not only on the applied stress but also on the accumulated thermal energy.

In addition, another feature was found during the tests, which is that the opened amount of the microcrack did not vary with an increase of the thermal and mechanical coupled loadings because the coating and substrate had been damaged at other points, such as the sample boundary. Therefore, other failure models may occur in the conducting polythiophene coating-substrate structure. Subsequently, this means that it is possible to improve the cracking resistance of conducting polythiophene coating when the agglomerative grain of the polymer is fine and small enough. Another defect was found in the vicinal fine regions and it is highly dependent on the processing conditions, not only the conductivity but also the thermal-mechanical properties of the conducting polythiophene coating-substrate structure [34].

The SEM *in situ* results showed that the characteristics of the crack initiation and propagation of conducting polythiophene coating appear to be complex. The failure models of the coating-substrate structure under thermal-mechanical loadings mainly represent that the thermal loading affects on the interface microcrack initiation and slight propagation in the vicinal greater agglomerative grain of polymer, and the macro-crack can be continuously formed at the interfaces of the agglomerated polymer grains in the coating layer.

## 6. Evaluation Method of Interface Adhesion Strength and Failure Stress of Coating-Substrate Structure

Most estimation methods of stress in film/coating-substrate structure were based on the Stoney formula [41] and its ramifications, which were based on the curvature radius *R.* This parameter *R* (*R* = (ℓ^2^ +δ^2^) / 2δ) can also be indirectly measured by δ displacement parameter in SEM *in situ* bending tests. In addition, Asaro [109] and Wang *et al*. [39] independently reported how to estimate failure stress in thin films. For example, it is shown that when the free surface of thin film is unstable or thin enough the following relationship exists:

(19) $$\sigma_{f}=\sqrt{\frac{8G\gamma \pi }{\lambda_{c}(1+\kappa )}}$$

where the subscript *c* denotes the critical mean. *G*, *γ*, *λ_c_* are the shear modulus, surface energy and unstable surface critical wave length of thin film, respectively, and *κ* denotes that stress state of thin film, which *κ* =3–4*ν_f_* denotes the plane strain state and *κ* =(3–*ν_f_*)/(1+*ν_f_*) denotes the plane stress state. In addition, Allen HG [110,111] and White JR [65] also reported that the estimation method of stress in the film or coating layer as shown as following respectively:

(20) $$\sigma_{f}^{w}=k{\left(E_{f}E_{s}^{2}\right)}^{1/3}$$

(21) $$\sigma_{f}=\frac{E_{f}}{6R(1-\mu_{f})}\frac{1+4\alpha \beta +6\alpha^{2}\beta +4\alpha^{3}\beta +\alpha^{4}\beta }{\left(1+\alpha )(1+\beta \right)}$$

where the constant *k* is about 0.0014 for the single layer of thin film or coat and about 0.0009 for multi-layers of thin film or coatings [110,111]. The constants *α,β* denote *α* =*h_f_* / *h_s_*, $\beta =\frac{E_{s}(1-\mu_{f})}{E_{f}(1-\mu_{s})}$, respectively. In Equation (21), the stress in the thin film is closely related to the bend curvature radius [65].

Wang *et al*. [35–39] recently reported the cracking behaviors of thin Cu film-substrate structures. They discovered that unstable wave motion occurred in the surface film/coating layer and they obtained the relationship between the bending loadings and curvature displacements of thin film-substrate structures based on the SEM *in situ* observation tests. Therefore, according to the measurement parameters, the failure stress in thin films can be estimated based on different methods. For example, when the thin film thickness is 1 μm and the parameters of *λ_c_*=20μ*m*,*G* =46*GPa*, *γ* =1.83*J* / *m*^2^ ,*ν_f_* =0.364 are substituted into Equation (19), the failure stress can be estimated. All estimation data of the failure stresses are listed in Table 2.

Therefore, Equation (19) provides a method of estimating failure stress for film-substrate structures which are thin enough, especially when the thickness of the thin film is less than 1 μm. The threshold value of estimating failure stress is closely related to the fracture process of the thin film-substrate structure or the cracking behavior of the thin film. Based on the fracture process of the thin film-substrate structure, the critical failure stress may be quantitatively estimated by the nano scratch and SEM *in situ* observation tests. It causes the more errors that other estimation methods of critical failure stress based on the curvature radius of the thin film-substrate structure or beam. That is, the effects of free surface stability, composite stress state and interfacial strength on the cracking behavior of thin film-substrate structure have to be considered when the thin film is thin enough. At the same time, these results are also suit for the polymer thin film/coating-substrate structures.

## 7. Summary

Understanding the mechanical behaviors of thin film/coating-substrate constructs is indispensable for the kinds of practical applications of thin film/coatings used in industry. The investigations on the mechanical properties, the cracking behavior and dependable influence factors of polymer film/coating substrate structures play a key role for their design and applications. These properties of polymers are strongly dependent on their microstructure, synthesis method and doping processing. In this work, there are not only the presently selected views of the mechanics analysis from the abundant literature, but also included the authors’ recent works. These results indicate that the following conclusions can be made:

1. The island by island nature of the electrochemical deposition behavior of conducting polymer has been validated by surface morphological analysis of different deposited states. Therefore, with the increase of film/coating thickness, these micro defects in the film/coating layer can directly affect the mechanical properties of conducting polymers, especially the Young’s modulus parameter. This is because the micro defects near the surface layer could not bear the applied loading. These cross-section areas will influence the estimated reliability of the nominal stress. In addition, these micro defects easily cause the stress concentration to occur early fracture. To obtain a conducting polymer film/coating without micro defects, the critical or effective thickness of conducting polymer film/coating layer is not than 5 μm closed to the substrate based on the three-electrode system when the total film thickness is about 65 μm.
2. By doping small amounts of nano particles, filler or ionic particles into the matrix, the mechanical properties of a polymer can be improved. One of the reasons is that the microstructure varies with the doping amount, but there is an optimal result. The high strength of thin film/coating on substrates can be attributed partly to the fine microstructures found in the conducting polymers. Further work is required to understand in more detail how to control the cracking behavior in small dimensions and how to improve the particle/filler size and texture.
3. The free surface cracking behavior of conducting polymer film/coating, taking into account the factors of temperature and holding time, can describe how cracks occur easily at the interface of greater agglomerated grains based on *in situ* SEM observations. The cracks’ main propagation direction is about 90° tilted to the mechanical loading direction. However, the increment of crack open displacement is about from 2.80 to 3.12 μm under a holding time 35 min at 100 °C. The effective difference of thermal distensibility between polymer and metal substrate on the crack proportion is still not obvious.
4. As the film/coating-substrate structure, the interested problem in engineering applications is how to estimate the failure model and critical failure stress in the film/coating, especially for thin coating layers. In general, the estimation methods of failure stress in coating layers are based on the Stoney formula and its ramifications. However, with the increasing of hardness difference of substrate and film/coating, the effective detection of curvature radius *R* becomes more and more difficult. In addition, as there is the stress gradient in a bent curvature, the estimation method of critical failure stress causes more errors based on the curvature radius. Therefore, the estimation method of failure stress for thin film/coating-substrate structures should be expressed combining the SEM *in situ* observation technology and scratch testing methods.
5. Investigations on the relationship between microstructure and mechanical of conducting polymer are insufficient up to the present, especially the studies on the relationship between the microstructure’s formation mechanism and cracking behavior. These behaviors can be described quantitatively to a certain extent.

## Figures and Tables

**Table 1.** Influence of PEG (DEG) molecular weight on the properties of PPy films produced in mixed electronlytes.

**Electrolyte composition and temperature**	**IPA+5%BFEE**		
	+5%DEG at 0 °C	+5%PEG1000 at 15 °C*	+5%PEG400 at 0 °C
Thickness (μm)	37.50	68.00	42.00
Density (kg/m^3^)	1,533.00	1,472.00	1,464.00
Conductivity (S/cm)	41.60	41.20	75.40
Tensile strength (MPa)	60.30	30.10	62.60
Elongation (%)	6.38	4.47	4.72

Notes:

* The experiment was performed at 15 °C because of the low solubility of PEG1000 in IPA at lower temperatures.

**Table 2.** Typical failure stress of 1 μm thin film-substrate structure under four-point bending loading [39].

**Interfacial strength (MPa)**	**Elastic Mechanics***σ* = *M* /*W***(MPa)**	**Stroney [40] (MPa)**	**Asaro [15] (MPa)**	**Allen [105,106] (MPa)**	**White [62] (MPa)**	**Wang [39] (MPa)**
230	306.6	363.6	237.8	170	316.5	235
